# The adolescent football mind: how age and playing position influence competitive state anxiety, self-confidence, and cognitive interference in Indian footballers

**DOI:** 10.3389/fspor.2025.1695658

**Published:** 2025-12-16

**Authors:** Debabrata Chatterjee, Santi Ranjan Dasgupta, Arkadeb Dutta

**Affiliations:** 1 Sports Neuroscience Laboratory, Department of Sports Science and Yoga, Ramakrishna Mission Vivekananda Educational and Research Institute (RKMVERI), Belur Math, India; 2 West Bengal State Council of Sports, Department of Youth Services and Sports, Govt. of West Bengal, Kolkata, India; 3 East Bengal Football Club, Maidan Tent, Kolkata, India; 4 Department of Biomedical Science & Technology, School of Biological Sciences, Ramakrishna Mission Vivekananda Educational and Research Institute (RKMVERI), Narendrapur, India

**Keywords:** competitive state anxiety, somatic anxiety, cognitive anxiety, cognitive interference, adolescence, Indian football, grassroots football, developmental psychology

## Abstract

**Introduction:**

Elevated competitive state anxiety, performance-interfering thoughts, and low self-confidence can impair coping ability to stress, and deteriorate athletic performance. Exaggerated competitive state anxiety is the manifestation of an athlete's negative appraisal of their own performance and fear of failure. Very little is known about the susceptibility to these psychological factors in adolescent athletes at grassroots football. The present study aimed to examine differences in competitive state anxiety, self-confidence, and cognitive interference scores between mid- and late adolescent Indian players and those with different playing positions. The inter-relationship between the age, playing experience [PE], cognitive state anxiety [CA], somatic state anxiety [SA], cognitive interference [CI], and self-confidence [SC] was also examined.

**Methods:**

Out of the ninety-one young (age range: 14–20 years), male footballers recruited from an elite football academy through convenience sampling, eighty-three (age: 17.08 ± 1.56 years; positions: goalkeepers = 12, defenders = 17, midfielders = 44, strikers = 10) completed the study. The original Revised Competitive State Anxiety Inventory (CSAI-2R) questionnaire and the Thought Occurrence Questionnaire for Sport (TOQS) were self-administered during intramural competition in mid-adolescent (MA, *n* = 50) and late-adolescent (LA, *n* = 33) players. Non-parametric correlation, Mann–Whitney *U*, and Kruskal–Wallis statistical tests were conducted.

**Results:**

Age was positively associated with PE (*τ* = +0.3, *p* < 0.001) and SC (*τ* = +0.16, *p* < 0.05); and negatively associated with CA (*τ* = −0.2, *p* < 0.05), SA (*τ* = −0.2, *p* < 0.05), thoughts of escape [ToE (*τ* = −0.19, *p* < 0.05)], situation-irrelevant thoughts [SIT (*τ* = −0.19, *p* < 0.05)], and performance worries [PW (*τ* = −0.15, *p* < 0.05)], CI (*τ* = −0.2, *p* < 0.001). The SA and CA were significantly higher in the MA than in LA (*p* < 0.01), as well as CI (*p* < 0.001). There was a significant effect of playing position only on CA scores (*p* < 0.05). *post-hoc* analysis found a significantly higher level of CA in strikers than the goalkeepers (*p* < 0.05) and midfielders (*p* < 0.05). PE did not show any significant association with these CSAI-2R and TOQS parameters.

**Discussion:**

Mid- adolescent players exhibited significantly lower SC and elevated SA, CA, and CI. The lower susceptibility to SA, CA, and CI was associated with increasing age and not with the PE. Adolescence in athletes is a critical phase for physiological, cognitive, and psychological maturation. Our findings emphasized the need for personalized psychological support in building mental resilience from a young age to cope with competitive pressure.

## 1 Introduction

Elite-level performance at the competitive stage in football not only hinges on physical and technical skills but also on psychological factors. A negative psychological state can deteriorate performance by causing errors in decision-making and can impair higher-order cognitive abilities required for accurate sports-specific motor execution (1, 2). A higher level of state anxiety in competition creates interference in executing set performance goals and maintaining self-confidence in athletes. Competitive anxiety emerges from a player's negative expectations and appraisal of their performance in a situation perceived as adverse (3–5). Hence, players experiencing elevated competitive state anxiety find it increasingly difficult to cope with the pressure and allocate mental resources meaningfully at a crucial stage of a game. In modern-day football, monitoring and regulating competitive state anxiety, improving coping ability, self-confidence, and optimal utilization of cognitive resources to enhance performance have become crucial aspects.

Cognitive anxiety and somatic anxiety are the two subcomponents of competitive state anxiety (4, 6). Cognitive anxiety is the mental element characterized by negative self-appraisal and expectations, rumination over irrelevant thoughts, low self-confidence, performance worries, and fear of failure (7, 8). These unpleasant thoughts and emotions can impair the ability to focus, disrupt the attentional shift between relevant tasks (attentional shifting), weaken the attentional control to resist interference from task-irrelevant stimuli (inhibitory control), and allocate working memory capacity to relevant tasks (9, 10). The impairment in attentional allocation ultimately causes performance deterioration (11). The intrusion of task-irrelevant cognitions and external distractions that limit working memory and other mental resources available for decision-making is referred to as cognitive interference (12, 13). Cognitive interference consumes cognitive resources that would otherwise be used for situation-relevant task-processing activities and hence, causes performance decline (14).

The somatic component of state anxiety is the physiological element comprising increased sympathetic activation, triggering arousal (7, 15). However, an overshooting somatic anxiety level causes a feeling of nervousness, heightens muscle tension, increases heart rate, and breathing difficulty that can lead to performance errors (5). Cognitive and somatic anxiety independently modulate the performance, and athletes who experience high levels of these elements may have low self-confidence, fall sick repeatedly, face sleeping problems, show aggressive behaviours, and drop out of sport (15, 16). Thus, their degree of intensification may predict whether athletes can emerge as winners or losers (2, 17, 18).

Over the years, assessing state anxiety levels in football players has gained importance (19). This is because state anxiety is not a stable personality trait but rather a player's momentary mental state and a decisive factor of a team's overall performance (19). A player with suddenly overpouring, negative thoughts and expectations during a match finds it difficult to maintain focus and cope with the challenges from the opponent team (20, 21). Interestingly, the brain areas that are activated during state anxiety are different from trait anxiety (22). This has emphasized the need for state-dependent targeted psychological management to maintain self-confidence and optimize the flow of cognitive resources on the task. The competitive state anxiety previously studied in professional footballers from different countries showed a common trend of reduction with increasing playing level and accumulated game experience (4, 23). It might be due to the increasing experience that players have learnt to manage stress and maintain cognitive resources. Several psychological facets, including self-confidence, attitudinal control, negative and positive coping control, visuo-imaginative control, and motivational level, may have contributed to maintaining a seamless flow and execution of skills for optimal performance (4, 19).

However, there is a paucity of information on state anxiety and its management in future sporting talents at grassroots football. A recent study has shown that young Spanish footballers in their adolescence exhibited greater competitive state anxiety and lower self-confidence and flow than older players (4). Mid- and late adolescence mark a critical developmental window for refining sports-related technical competencies and psychological coping mechanisms (24, 25). Neurodevelopmental processes during adolescence include synaptic pruning and increased myelination in the higher-level cognitive domains of the brain (26). They enhance cognitive and executive functions crucial for adapting to dynamic game situations (26). The adolescent stage is also characterized by heightened sensitivity to social evaluation and peer comparison (25). Without proper management, these factors intensify in young athletes and develop performance-related anxiety (25). Since psychological factors have social, cultural, and ethnic influences, studies targeting different populations and ethnicities may shed a better understanding of coping with competitive state anxiety in adolescence across cultures and ethnicities. In the context of Indian football, understanding the association between competitive state anxiety with the developmental trajectories of adolescence is crucial in developing coping skills and improving performance (25, 26).

In addition, the role of playing positions on state anxiety and psychological coping in adolescence also needs attention. Football is a dynamic sport where players have different roles and responsibilities that vary with their playing positions (27). Positions where players frequently face relatively difficult challenges and expectations to overcome them may require delicate coordination between mental coping with pressure and motor execution at a younger age. For example, a sudden shift to attacking tactics by the opposition may demand a more focused, precise, and adequate response from defenders and goalkeepers (28). Such a momentary increase in cognitive workload may invoke higher competitive anxiety and cognitive interference in players (27, 29). Therefore, position or role-specific psychological assessments for identifying anxiety and interference levels are essential in adolescent footballers (27). However, the association between playing positions and competitive anxiety has led to mixed results. Few studies have reported different psychological profiles and competitive anxiety levels between playing positions (30, 31); however, there are also reports of no changes in competitive anxiety with playing positions in high-level players (4, 23, 32, 33).

The mutual interaction between maturity, playing experience, cognitive, and psychological factors determines the performance outcome of an athlete (34). Understanding the relationship between these factors in professional Indian adolescent footballers could help implement proper strategies to prevent choking under pressure and optimize performance. The present study aims to explore the association between the competitive state anxiety levels, self-confidence, cognitive interference, chronological age, experience, and playing positions in Indian adolescent footballers. Playing experience and age are important factors in managing anxiety during competitive events (4, 23). In a study on youth volleyball players, experience was found to mediate the relation between age and sports-specific decision-making (35). Whether the playing experience has a mediating effect on the relationship between age and competitive state anxiety, self-confidence, and cognitive interference in adolescent footballers is not known. Therefore, the first hypothesis of the study is that competitive state anxiety and cognitive interference will be negatively associated with age and playing experience, and self-confidence will show a positive relation with age and experience. Age and playing experience would also have a positive association. Mid- and late adolescence development displays distinct neurobiological and psychosocial processes that influence maturity in action and behavior (26, 36). Our second hypothesis was that mid- and late adolescent players would exhibit differing levels of competitive state anxiety, self-confidence, and cognitive interference. The performance intensity, enhanced responsibilities, and expectations from players may vary with their playing positions (16). Inability to deliver in decisive moments of the game in the continuously demanding positions may sometimes cause coping problems. Hence, the third hypothesis of the study is that state anxiety levels, self-confidence, and cognitive interference will differ with the playing positions.

## 2 Materials and methods

### 2.1 Study design

A comparative observational study design was employed to investigate any significant differences in competitive anxiety and cognitive interference levels between early and late adolescence, as well as between different playing positions. The associations of age with playing experience, and their relationship with the competitive state anxiety, negative, irrelevant performance-interfering thoughts, were also examined. Eligibility criteria included male footballers with self-reported normal vision and no neurological deficit.

### 2.2 Participants

Ninety-one young, male football players affiliated with an elite football academy (accredited by the All India Football Federation) were registered for this study through convenience sampling. The weekly training schedule of the participants included five field-based and one gym-based training session. Eight athletes were excluded due to a lack of continuity in the middle of the study. Finally, the tests were conducted on 83 athletes between 14 and 20 years of age (mean ± SD: 17.08 ± 1.56 years), BMI (mean ± SD: 18.99 ± 1.67 kg/m^2^), including 12 goalkeepers, 17 defenders, 44 midfielders, and 10 strikers. The age range was appropriate for identifying and training future elite athletes (37). This period is also characterized by intense performance anxiety in adolescent players in competitive and organized sports (38). The athletes were categorized into two groups: mid-adolescent (MA, *n* = 50) and late adolescent (LA, *n* = 33). According to Brown et al. (39), 14–16 years mark MA, and 17–20 years are LA. The study procedures followed the guidelines of the Declaration of Helsinki and were approved by the Institutional Ethics Committee (IEC/2023/07/ SSY01). Before the study, participants and their parents were informed about the purpose and benefits of the investigation. Written consents signed by the legal guardians of the participants were collected.

### 2.3 Procedures

The demographic characteristics and personal information of the subjects, including age, playing experience, and playing position (goalkeeper, defender, midfielder, or striker), were recorded using a data form. Some participants have earlier played in either or both state and national level competitions in the U-15 to U-19 age categories. The present study considered playing experience as the total amount of time in years a participant has played under the supervision of accredited coaches in grassroots football academies. The subjects were fluent in English; hence, the original English versions of the Revised Competitive State Anxiety Inventory (CSAI-2R) questionnaire and the Thought Occurrence Questionnaire for Sport (TOQS) were used in paper form (6, 13). Competitive state anxiety was evaluated in the athletes belonging to a macro-cycle under the supervision of the same group of coaches. The ratings were taken during the intramural competition for the academy team selection to participate in the different age category national leagues.

CSAI-2R was completed 1 h before the start of an intramural match, and participants fully completed the inventory under 10–15 min. The instrument application time-point is considered acceptable since it did not interfere with the player's mental preparation (40). TOQS was self-administered based on the most recent concluded intramural match. The factorial validity of this scale has been supported in both adult (13) and youth sport populations (41).

CSAI-2R is a 17-item revised version of the Competitive State Anxiety Inventory. CSAI-2R is a validated and reliable instrument to measure competitive anxiety in sports settings (6, 8). The instrument has three subscales to assess competitive anxiety, namely somatic anxiety (7 items), cognitive anxiety (7 items), and self-confidence (7 items). CSAI-2R uses a 4-point Likert scale (not at all =0, somewhat =1; moderately =2, very much so =3) for each item. Somatic anxiety (SA) is the summated scores of item numbers 1, 4, 6, 9, 12, 15, and 17; cognitive anxiety (CA) is assessed by combining the scores of item 2, 5, 8, 11, 14; and self-confidence is measured (SC) by summing up the scores of the items 3, 7, 10, 13, 16. The score range of SA, CA, and SF is 0–21, 0–15, and 0–15, respectively.

TOQS was used to subjectively measure cognitive interference experienced by the footballers in the intramural match (13). It is a 17-item self-administered questionnaire comprising three subscales, namely performance worries (6 items), situation-irrelevant thoughts (5 items), and thoughts of escape (6 items). The score of each item was calculated from a 7-point Likert scale. The score range for performance worries (PW, items 3, 6, 9, 12, 15, 17), situation-irrelevant thoughts (SIT, items 2, 5, 8, 11, 14), and thoughts of escape (ToE, items 1, 4, 7, 10, 13, 16) is 1–42, 1–35, 1–42, respectively. Cognitive interference (CI) is the total score of the 17 items. Mean values of PW, SIT, ToE, and CI scores were recorded.

### 2.4 Statistical analysis

All the statistical analyses were performed in RStudio statistical software and programming package (version 2025.05.0 + 496, Posit Software, PBC). Significance was set at *p* < 0.05. Kolmogorov–Smirnov test was conducted to test the normal distribution of the data. Non-parametric statistical tests were conducted on the data when the normality assumption was not met. Kendall's correlation coefficient (*τ*) analysis was performed between age, playing experience, CSAI-2R scores (somatic anxiety, cognitive anxiety, self-confidence), and TOQS scores (performance worries, situation-irrelevant thoughts, thoughts of escape, and cognitive interference scores). The magnitude of the correlation coefficient was considered small (0.1 ≤r < 0.3), moderate (0.3 ≤r < 0.5), large (0.5 ≤r < 0.7), very large (0.7 ≤r < 0.9), and nearly perfect (r ≥ 0.9). A Mann–Whitney U test was performed to find any significant differences in the CSAI-2R and TOQS scores between the age groups. Effect size was expressed as Glass rank-biserial coefficient (rg). Values approaching towards +1 or −1 from 0 indicate larger differences in proportion between favorable and unfavorable signed ranks of measuring variable (42). A Kruskal–Wallis test was conducted to find any significant changes in the cognitive anxiety, somatic anxiety, self-confidence and cognitive interference scores between the playing positions. If Kruskal–Wallis test was significant, Dunn test was used as a *post-hoc* analysis to determine the level of differences between the unequal groups and *p*-values adjusted with Benjamin-Hochberg (B-H) method. To understand the magnitude of pairwise differences, the effect size was calculated as Cliff's delta (CD). CD expresses the strength of non-overlap between two groups ranging between −1 to +1. CD away from 0 on either direction indicates the increasing degree of non-overlap between the groups. The effect size for rg and CD was considered to be small between 0.1 to <0.3, medium between 0.3 to <0.4, and large above 0.4 according to Lovakov and Agadullina (43).

## 3 Results

The descriptive data of age, BMI, and playing experience, and the scores of CSAI-2R and TOQS variables are provided in Table 1. The BMI values in both the groups are within the normal range of the Indian population (44).

**Table 1:** Descriptive statistics of physical characteristics, experience, CSAI-2R and TOQS scores.

Parameters	Total (*n* = 83)	MA (*n* = 50)	LA (*n* = 33)
Age (years)	17.08 ± 1.58	16.02 ± 0.65	18.7 ± 1.15
BMI (kg/m^2^)	18.99 ± 1.67	18.81 ± 1.39	19.27 ± 2.05
PE (years)	3.8 ± 2.76	3.1 ± 2.37	4.9 ± 3.02
CSAI-2R-SA	9.8 ± 2.76	10.5 ± 2.91	8.7 ± 2.18
CSAI-2R-CA	11.6 ± 2.84	12.4 ± 2.73	10.6 ± 2.69
CSAI-2R-SC	16.0 ± 3.41	15.4 ± 3.56	16.9 ± 3.0
TOQS-ToE	1.62 ± 0.59	1.72 ± 0.64	1.46 ± 0.48
TOQS-SIT	1.65 ± 0.85	1.84 ± 0.97	1.36 ± 0.51
TOQS-PW	2.79 ± 0.92	3.0 ± 0.93	2.46 ± 0.80
TOQS-CI	2.04 ± 0.59	2.21 ± 0.62	1.79 ± 0.44

Values expressed as Mean ± SD.

PE, personal experience; CSAI-2R, competitive state anxiety inventory revised; TOQS, thought occurrence questionnaire; SA, somatic anxiety; CA, cognitive anxiety; SIT, situational irrelevant thoughts; PW, performance worries; CI, cognitive interference.

Kendall's *τ*, showing the strength of correlation, is presented in Table 2. A positive and significant association of age with PE (effect size: moderate) and SC (effect size: small) was observed. Consistent with the first hypothesis, the association of age with CA, SA, ToE, SIT, PW, and CI was significant and negative with a small effect size. But PE did not show any significant association with these CSAI-2R and TOQS parameters. A significant negative association with small effect was noticed between the SA and SC, whereas SA exhibited a positive and significant correlation with CA, ToE, PW, SIT, and CI with medium effect size. A similar trend in association and effect size of CA with SC, and between CA and ToE, SIT, PW, and CI was observed. The negative association of SC was significant with SIT, PW, and CI with a small effect size, but not with the ToE. CI showed a positive association with ToE, SIT, and PW with a large effect size. ToE, SIT and PW showed positive mutual association with effect size ranging from small to medium.

**Table 2:** Correlations (Kendall's *τ*) between age, experience and the competitive values of the psychological variables in young football players (*n* = 83).

	Age	PE	SA	CA	SC	ToE	SIT	PW	CI
**Age**	−								
**PE**	0.3***	−							
**SA**	−0.2*	−0.037	−						
**CA**	−0.2*	−0.035	0.4***	−					
**SC**	0.16*	0.011	−0.16*	−0.3***	−				
**ToE**	−0.19*	.0035	0.3***	0.3***	−0.11	−			
**SIT**	−0.19*	−0.11	0.31**	0.24**	−0.2**	0.22**	−		
**PW**	−0.15*	−0.057	0.37***	0.43***	−0.2**	0.31***	0.28***	−	
**CI**	−0.2**	−0.08	0.4***	0.4***	−0.2**	0.5***	0.5***	0.7***	−

PE, personal experience; CSAI-2R, competitive state anxiety inventory revised; TOQS, thought occurrence questionnaire; SA, somatic anxiety; CA, cognitive anxiety; SIT, situational irrelevant thoughts; PW, performance worries; CI, cognitive interference.

Significance level:

* *p* < 0.05.

** *p* < 0.01.

*** *p* < 0.001.

**Figure 1:**
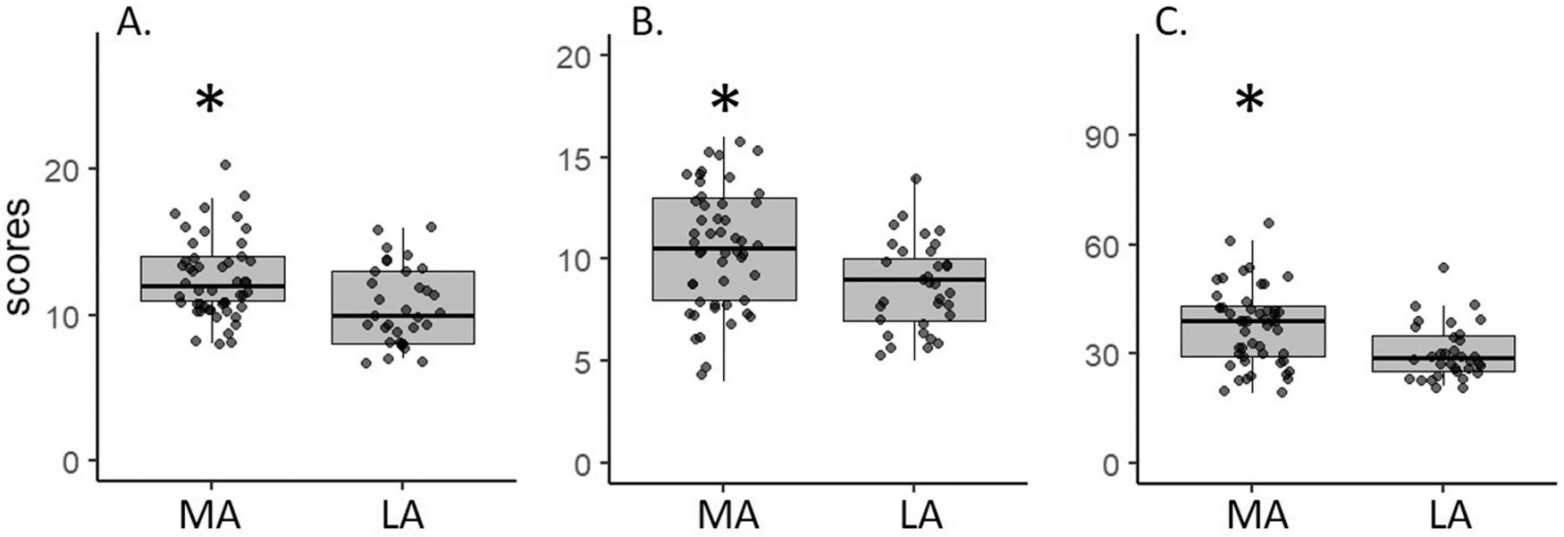
The effect of age group differences (MA vs. LA) in **(A)** somatic anxiety (SA), **(B)** cognitive anxiety (CA), and **(C)** cognitive interference (CI) scores. The boxes indicate the interquartile range with the median (bold black line inside each box). Whiskers indicate the minimum and maximum scores. Each dot represents the score of the athletes. MA, mid- adolescence; LA, late adolescence; * *p* < 0.05.

The SA and CA in CSAI-2R were significantly higher in the MA athletes in comparison to the LA athletes (SA: U = 1,130.5, *p* = 0.004; CA: U = 1,124.5, *p* = 0.005). However, SC was not significant between the MA and LA athletes (U = 617, *p*-value = 0.052) indicating partial fulfillment of the second hypothesis. The SA and CA scores were lower in LA than MA, with moderate effect size of −0.37 and −0.36 respectively. CI score of the TOQS was significantly lower in the LA group in comparison to the MA group with a large effect size of −0.42 (CI: U = 1,170.5, *p* = 0.001) (Figure 1).

**Figure 2:**
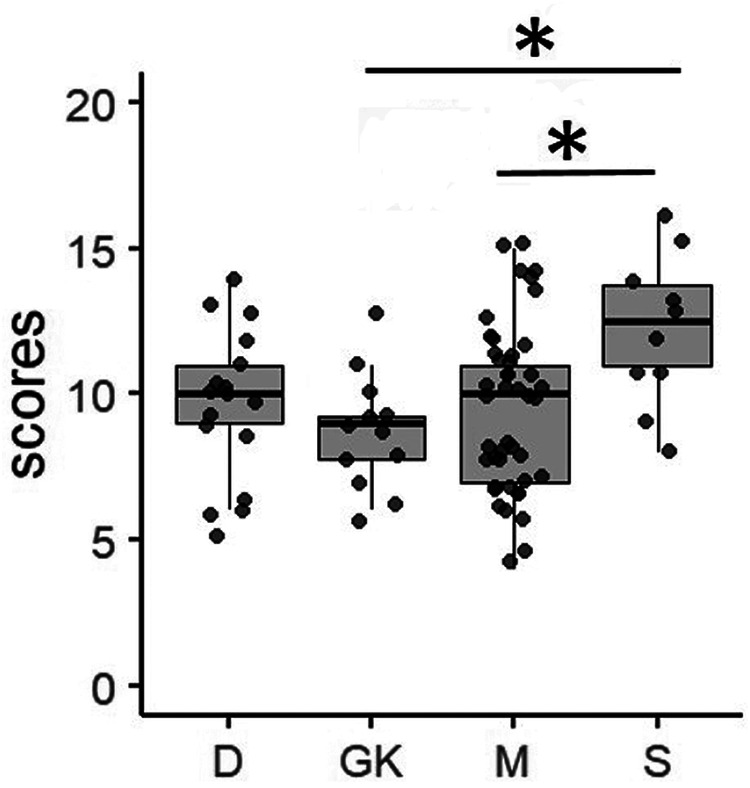
The effect of playing position (D, GK, M, S) on cognitive anxiety (CA) scores. The boxes indicate the interquartile range with the median (bold black line inside each box). Whiskers indicate the minimum and maximum scores. Each dot represents the score of the athletes. D, defender; GK, goalkeeper; M, midfielder; S, striker; * adjusted-p (B-H method) < 0.05.

There was a significant effect of playing position only on CA scores (Kruskal–Wallis chi-squared = 9.0404, df = 3, *p* = 0.029). *post-hoc* analysis found a significantly higher level of CA in strikers than the goalkeepers [B-H adjusted *p* = 0.012; CD = −0.7 (large effect size)] and mid-fielders [B-H adjusted *p* = 0.016; CD = 0.52 (large effect size)]. No significant difference was found between the strikers and the defenders [B-H adjusted *p* = 0.03; CD = 0.17 (small effect size)] [Figure 2]. It was also found that the playing position of the athletes has no significant effect on SA (Kruskal–Wallis chi-squared = 4.9069, df = 3, *p* = 0.18), SC (Kruskal–Wallis chi-squared = 1.6109, df = 3, *p* = 0.66), and CI (Kruskal–Wallis chi-squared = 3.6015, df = 3, *p* = 0.31) indicating partial-fulfillment of the third hypothesis.

## 4 Discussion

The present study explored the levels of competitive state anxiety, self-confidence, and cognitive interference in the mid- and late adolescent Indian football players. The association between playing experience and chronological age, competitive state anxiety, and cognitive interference was tested. The first hypothesis which proposed a significant association between the age and playing experience and associations of age and playing experience with competitive state anxiety, self-confidence, and cognitive interference was partially fulfilled. The second hypothesis proposed differences in competitive state anxiety, self-confidence, and cognitive interference between the mid- and late adolescent players. Consistent with this hypothesis, the cognitive state anxiety, somatic state anxiety, and cognitive interference differed significantly between the mid- and late adolescent players. However, it is partially fulfilled, as self-confidence did not significantly vary between the groups. In the third hypothesis, all the measured psychological variables were proposed to vary with the playing positions. However, it was partially fulfilled due to the effect of playing position only on cognitive anxiety.

To the best of our knowledge, the present study is possibly the first to report the difference in competitive state anxiety and cognitive interference between the adolescent stages in young Indian footballers. Cognitive state anxiety and somatic state anxiety scores were higher in mid-adolescent players than late-adolescent players. A previous study on Spanish footballers showed similar findings, where 14–15 years age category players had greater somatic state anxiety than 16–18 years age category players (4). The same study found comparatively low competitive state anxiety in the group playing at the higher level. Playing level and the number of competitive matches played are also considered playing experience in football (23, 45–47). Previous studies have shown that players having these experiences have better coping ability under stress and reduced competitive anxiety levels (4, 23). With increasing age and accumulating experience, players become efficient in allocating focus and cognitive resources into the game while managing competitive anxiety (48).

The effect of playing experience on age-related changes in the competitive state anxiety levels and cognitive interference in the adolescent footballers has not been examined yet. In our study, the age of players showed a significant negative association with cognitive and somatic state anxiety and a positive association with the players' self-confidence. Previous studies have reported the same trend in football and other sports (4, 45, 49). We also found a significant positive association between playing experience and age. We further examined whether experience has any role in the relationship between age and the recorded psychological parameters in Indian youth players. A previous study on youth volleyball players has found that experience mediates the relationship between age and sports-specific cognition (35). Cognitive functions and decision-making in sports are impaired by elevated state anxiety levels and cognitive interference (12, 50). However, we did not find any significant association between playing experience and the competitive state anxiety and cognitive interference. Hence, the changes were associated with age-related maturity. The experience, counted as the time period (in years) a player has been under a coach's supervision in grassroots football, increases with age. The players underwent various forms of training, match participation, and conditioning during this period. These adolescent players lacked significant experience at the advanced competitive level. This might be a reason for the lack of any significant association between playing experience and the subjective feelings of state anxiety and cognitive interference during a competitive scenario. A better approach in future would be considering the number of matches played at the advanced level. So, we could not overrule the effect of other extrinsic or intrinsic factors mediating the relation between age and psychological parameters that were beyond the scope of the present study.

Negative expectations of performance, fear of failure arise in players under a competitive scenario (5). These task-irrelevant, intrusive thoughts can shift the focus from the game, leading to early burnout (51). We have estimated these cognitive interfering thoughts using TOQS, and found that cognitive interference and its components (ToE, SIT, PW) are positively associated with cognitive state anxiety and somatic state anxiety. Like cognitive anxiety and somatic anxiety, cognitive interference was negatively related to self-confidence. Our findings are consistent with the interference of flow and the lowering of self-confidence with increased anxiety levels in the footballers. The interfering thoughts have been observed to decrease with increasing age.

As discussed earlier, the age-related maturity, rather than years of training, is found to be the primary element for the reduction of competitive anxiety and cognitive interference levels in adolescent footballers. This reduction in competitive state anxiety and cognitive interference levels from mid- to late-adolescence can be explained as a natural psychophysiological process occurring due to critical developmental shifts. The prefrontal cortex of the brain regulates decision-making and impulse control (52). The connections in these areas are not fully developed in mid-adolescence but strengthen towards the late-adolescent period (53). In mid-adolescent age, elevated pubertal hormonal fluctuations interact and affect the brain functions, which in turn can cause an increase in emotional reactivity, leading to increased cognitive interference as well as anxiety levels (54). Late adolescence is characterized by an increase in gray matter volume and maturation of the frontal lobe, known as the seat of attentional regulation and stronger executive functioning (55). In late adolescence, athletes attain the cognitive functions to understand and remember complex strategies with fully matured perceptual-motor skills (39). These changes might facilitate coping ability and lower anxiety under stressful situations.

One of the objectives of this study was to examine the changes in the levels of cognitive state anxiety and cognitive interference based on playing position. Previous studies on competitive state anxiety levels relative to the playing positions of footballers have produced mixed results (4, 23, 30–33). While a few studies have found significant differences in the competitive state anxiety components and self-confidence in different positions (30, 31), most of the studies did not find any change (4, 23, 32, 33). We have found that the somatic component of the competitive state anxiety remained unchanged in the players at different playing positions, which might indicate that the physiological manifestation of stress was independent of playing positions in our study. However, the cognitive state anxiety level was higher in the strikers than in the goalkeepers, midfielders, and defenders. The difference was significant from the goalkeepers and midfielders. The probable reason is that the strikers might have faced difficulty maintaining focus on high-intensity activity, showing opponent-evading skills in high-stakes situations. In general, strikers usually perform the highest number of high-intensity sports-specific decision-making and actions (56). However, we found different results from the previous two studies, where the goalkeepers and the defenders exhibited higher cognitive state anxiety than others (30, 31). We explain this discrepancy from observation, as state anxiety is a temporary, more malleable, situation-specific psychological phenomenon (19). It is difficult to generalize state anxiety in an athlete due to its transient nature and based on perceived conditions as adverse. These stressors, including expectation to perform, fear of failure, and negative appraisal from the coaches, teammates, and spectators, can interfere in various ways with the functioning of sports-specific cognition and actions (57). Hence, we emphasize the need for individualized and daily psychological support to athletes as the state anxiety may vary with the spatial (position-specific) and temporal (time point in a game) nature of potential stressors, experience, and playing status. This is supported by the “Individual Zones of Optimal Functioning Model”, each individual has an optimal zone of arousal/anxiety where they can reach peak performance, and if their arousal/anxiety is outside of the zone (too low or too high) on any day, performance will decline on that day (58).

The major strength of the present study was examining the intensity of state anxiety, self-confidence, and interfering thoughts during competition in the adolescent Indian footballers at the grassroots. Most studies focused on psychological health in top-level athletes. However, emphasis should also be given to grassroots football for identification and nurturing of future talents. Adolescence in athletes is a crucial window for the development and maturation in physiological, cognitive, and psychological spheres (39). We tried to address this aspect and found that mid-adolescent footballers are more susceptible to stressful situations, reflected in their elevated state anxiety scores, frequent intrusive thoughts. The findings of our study emphasized the need for personalized psychological sessions and support in building mental resilience to excel in performance at a young age. Young athletes must learn about stress management to focus on their planned skills and abilities in adverse situations in a match. A previous study has found that footballers of Malay ethnic origin have higher cognitive state anxiety and lower somatic state anxiety than the footballers of Indian ethnic origin. The course of psychological development in a younger age may also vary across cultures, leading to different coping strategies with stress. Anxiety, self-confidence, and cognitive interference scores could not be generalized across cultures and ethnicities. Therefore, to the best of our knowledge, we targeted two adolescent groups of Indian footballers and compared their levels of competitive state anxiety, self-confidence, and cognitive interference.

One major limitation of our study was the limited number of players selected from a three-star football academy. Including the neighboring accredited football academies, nurturing young talents, could have increased the sample size to decipher the psychological identity and variations of the footballers linked with the demography of that region. Due to the lack of the required number of samples in some playing positions, the study could not examine the influence of adolescent stages on the psychological profiles at different playing positions. This was also a limitation of the study. Another limitation was not measuring experience in terms of the number of competitive matches played by an athlete. This might have elucidated a mediating effect of playing experience on the relationship between age and the psychological factors in footballers.

## 5 Conclusion

Psychological factors such as anxiety, self-confidence, and cognitive interference impact sports performance. Athletes with lower self-confidence and higher anxiety levels often find it difficult to cope under pressure and choke. A complex interaction between intrinsic and extrinsic factors underlies the outcome of competitive performance. Our findings indicate that middle adolescent players are particularly vulnerable to anxiety and interference, and that this vulnerability is influenced by age and maturity rather than just playing experience. The strikers have a greater cognitive anxiety level than those in other playing positions, emphasizing the importance of position-specific psychological support at the individual level for coping with psychological stress and enhancing performance during competitive scenarios. Our results emphasized the application of age and position-specific psychological sessions and personalized mental support in building mental resilience to excel in performance from a young age. Coaches and sports science practitioners may consider integrating psychological sessions in mid- and late adolescent footballers during the pre-competitive and competitive phases.

## Data Availability

The original contributions presented in the study are included in the article, further inquiries can be directed to the corresponding author.
